# Integrated thick-film nanostructures based on spinel ceramics

**DOI:** 10.1186/1556-276X-9-149

**Published:** 2014-03-26

**Authors:** Halyna Klym, Ivan Hadzaman, Oleh Shpotyuk, Michael Brunner

**Affiliations:** 1 Lviv Polytechnic National University, 12 Bandera str, Lviv 79013, Ukraine; 2 Drohobych State Pedagogical University, 24 Ivan Franko str, Drohobych 82100, Ukraine; 3 Lviv Institute of Materials of Scientific Research Company ‘Carat’, 202 Stryjska str, Lviv 79031, Ukraine; 4 Institute of Physics of Jan Dlugosz University, 13/15, al. Armii Krajowej, Czestochowa 42201, Poland; 5 Fachhochschule Köln/University of Applied Sciences, 2 Betzdorfer Strasse, Köln 50679, Germany

**Keywords:** Spinel, Ceramics, Thick films, Integrated nanostructure, Simultaneous measurements

## Abstract

Integrated temperature-humidity-sensitive thick-film structures based on spinel-type semiconducting ceramics of different chemical compositions and magnesium aluminate ceramics were prepared and studied. It is shown that temperature-sensitive thick-film structures possess good electrophysical characteristics in the region from 298 to 358 K. The change of electrical resistance in integrated thick-film structures is 1 order, but these elements are stable in time and can be successfully used for sensor applications.

## Background

Nanostructured functional spinel-type ceramics based on magnesium aluminates and mixed transition metal manganites are known to be widely used for temperature and humidity measurement [1-5]. But their sensing functionality is restricted because of bulk performance allowing no more than one kind of application.

A number of important problems connected with hybrid microelectronic circuits, multilayer ceramic circuits, temperature sensors, thermal stabilizers, etc. require such resolution, when not bulk (e.g., sintered as typical bulk ceramics), but only the thick-film performance of electrical components (possessing the possibility to group-technology route) is needed [5]. The well-known advantages of screen printing technology revealed in high reproducibility, flexibility, attainment of high reliability by glass coating, as well as excellent accuracy, yield, and interchangeability by functional trimming are expected to be very attractive now for new-generation sensing electronics [6]. No less important is the factor of miniaturization for developed thick-film elements and systems, realized in a variety of their possible geometrical configurations. Thus, the development of integrated nanostructured thick films based on spinel-type compounds for multifunctional temperature-humidity sensors is a very important task [6-8].

To fabricate the integrated temperature-humidity thick-film sensors, only two principal approaches have been utilized, they being grounded on temperature dependence of electrical resistance for humidity-sensitive thick films and/or on humidity dependence of electrical resistance for temperature-sensitive thick films. The first approach was typically applied to perovsite-type thick films like BaTiO_3_[9]. Within the second approach grounded on spinel-type ceramics of mixed Mn-Co-Ni system with RuO_2_ additives, it was shown that temperature-sensitive elements in thick-film performance attain additionally good humidity sensitivity [10]. Despite the improved long-term stability and temperature-sensitive properties with character material *B* constant value at the level of 3,000 K, such thick-film elements possess only small humidity sensitivity. This disadvantage occurred because of relatively poor intrinsic pore topology proper to semiconducting mixed transition metal manganites in contrast to dielectric aluminates with the same spinel-type structure.

The thick-film performance of mixed spinel-type manganites restricted by NiMn_2_O_4_-CuMn_2_O_4_-MnCo_2_O_4_ concentration triangle has a number of essential advantages, non-available for other ceramic composites. Within the above system, one can prepare the fine-grained semiconductor materials possessing p^*+*^-type (Cu_0.1_Ni_0.1_Mn_1.2_Co_1.6_O_4_) and p-type of electrical conductivity (Cu_0.1_Ni_0.8_Mn_1.9_Co_0.2_O_4_). Prepared thick-film nanostructures involving semiconductor NiMn_2_O_4_-CuMn_2_O_4_-MnCo_2_O_4_ and insulating (i-type) MgAl_2_O_4_ spinels can be potentially used as simultaneous thermistors and integrated temperature-humidity sensors with extremely rich range of exploitation properties.

The aim of this work is to develop the separate temperature- and humidity-sensitive thick-film nanostructures based on spinel-type ceramics, in which the semiconducting thick films based on NiMn_2_O_4_-CuMn_2_O_4_-MnCo_2_O_4_ ceramics are used not only as temperature-sensitive layers but also as conductive layers for humidity-sensitive thick films based on MgAl_2_O_4_ ceramics.

## Methods

Previously studied and selected samples of Cu_0.1_Ni_0.1_Co_1.6_Mn_1.2_O_4_, Cu_0.1_Ni_0.8_Co_0.2_Mn_1.9_O_4_, and MgAl_2_O_4_ spinel ceramics with optimal structural properties [11-18] were used for the preparation of temperature- and humidity-sensitive thick-film layers.

Temperature-sensitive ceramics were prepared by a conventional ceramic processing route using reagent grade cooper carbonate hydroxide and nickel (cobalt) carbonate hydroxide hydrates [11]. The Cu_0.1_Ni_0.1_Co_1.6_Mn_1.2_O_4_ ceramics were sintered at 1,040°C for 4 h and Cu_0.1_Ni_0.8_Co_0.2_Mn_1.9_O_4_ ceramics at 920°C for 8 h, 1,200°C for 1 h, and 920°C for 24 h [19-23]. As a result, we obtained single-phase spinel Cu_0.1_Ni_0.1_Co_1.6_Mn_1.2_O_4_ ceramics (temperature constant *B*_25/85_ = 3,540 K) and Cu_0.1_Ni_0.8_Co_0.2_Mn_1.9_O_4_ ceramics (*B*_25/85_ = 3,378 K) with additional NiO phase (10%) [12].

The bulk MgAl_2_O_4_ ceramics were prepared via conventional sintering route as was described in more details elsewhere [13-18]. The pellets were sintered in a special regime with maximal temperature *T*_s_ = 1,300°C for 5 h.

Temperature-sensitive Cu_0.1_Ni_0.1_Co_1.6_Mn_1.2_O_4_/Cu_0.1_Ni_0.8_Co_0.2_Mn_1.9_O_4_-based pastes were prepared by mixing powders of basic ceramics (72.8% of sintered bulk ceramics were preliminarily destroyed, wet-milled, and dried) with ecological glass powders (2.9%) without PbO, inorganic binder Bi_2_O_3_ (2.9%), and organic vehicle (21.4%). The next content was used for the preparation of humidity-sensitive thick-film pastes: MgAl_2_O_4_-based ceramics (58%), Bi_2_O_3_ (4%), ecological glass (8%), and organic vehicle (30%).

**Figure 1:**
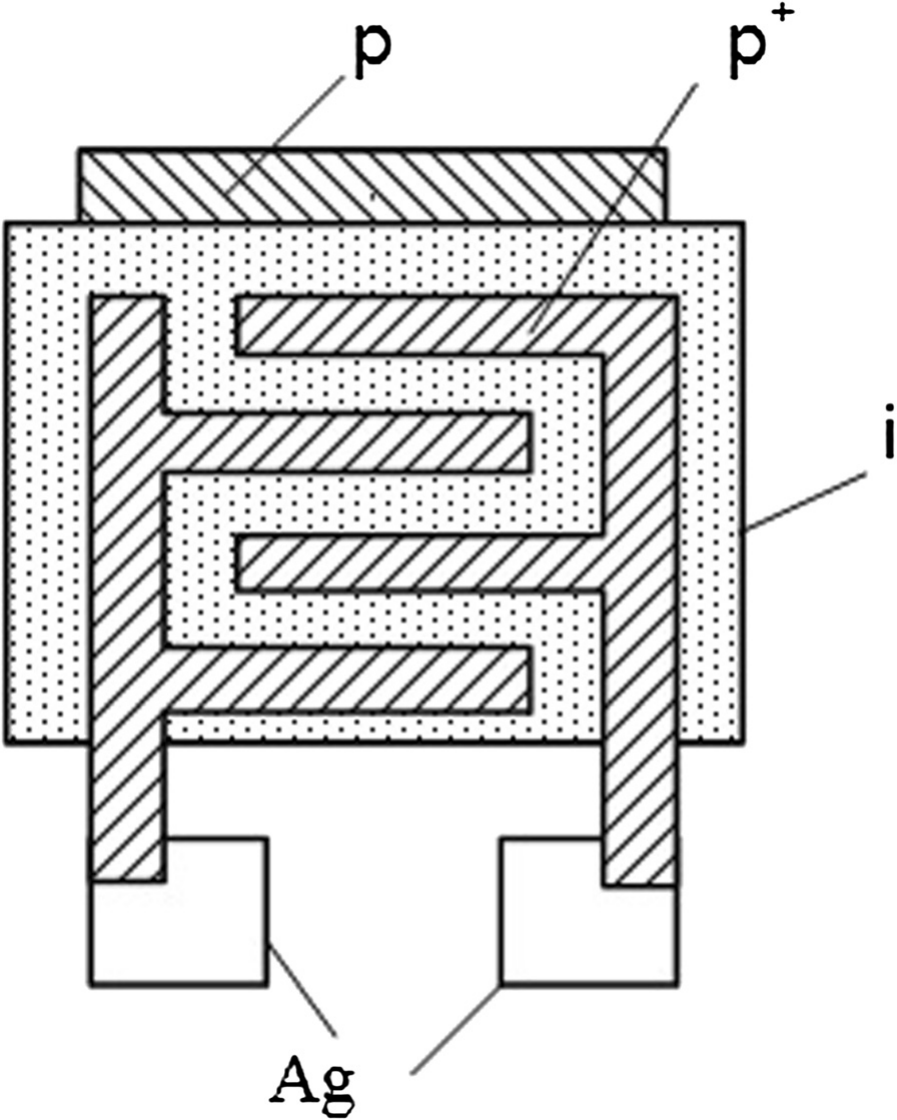
**Topological scheme of integrated thick-film p-i-p**^**+ **^**structure.**

The pastes were printed on alumina substrates (Rubalit 708S, CeramTec, Plochingen, Germany) using a manual screen printing device equipped with a steel screen. Then, thick films were sintered in PEO-601-084 furnace at 850°C [20,23]. The insulating (i-type) paste in two layers was printed on temperature-sensitive (p-type) thick-film layer previously formed on alumina substrate. In contrast to previous works [21,23], the p^+^-conductive paste was formed on humidity-sensitive i-type layer as conductive layer. Then, these structures were sintered in the furnace. The topological scheme of integrated p-i-p^+^ thick-film structure is shown in Figure 1.

The microstructure of the sintered temperature-sensitive ceramics was probed using an electron microscope JSM-6700 F (JEOL Ltd., Akishima, Tokyo, Japan), cross-sectional morphology of the samples being tested near the surface (0- to 70-μm depth) and chip centers. Scanning electron microscopy (SEM) investigations for bulk humidity-sensitive ceramics and thick-film structures were performed using LEO 982 field emission microscope (Carl Zeiss AG, Oberkochen, Germany).

The pore size distribution of bulk semiconductor and dielectric ceramics in the region from 2 to 1,000 nm was studied using Hg-porosimetry (POROSIMETR 4000, CARLO ERBA STRUMENTAZIONE, Hofheim am Taunus, Germany).

The electrical resistance of thermistor thick films was measured using temperature chambers MINI SUBZERO, Tabai ESPEC Corp., Japan, model MC-71 and HPS 222. The humidity sensitivity of thick-film structures was determined by measuring the dependence of electrical resistance *R* on relative humidity (RH) of the environment. The electrical resistance was measured in the heat and humidity chamber PR-3E (Tabai, Osaka, Japan) at 20°C in the region of RH = 20% to 99%. The electrodes were attached to connecting cables of M-ohmmeter at fixed current frequency of 500 Hz (with the aim of avoidance of polarization of adsorbed water molecules). In addition, the degradation transformation at 40°С and RH = 95% for 240 h was carried out in order to study sample stability in time. The maximal overall uncertainties in the electrical measurements did not exceed approximately ± (0.02 to 0.04) MΩ in electrical resistance. The confidence interval in RH measuring bar restricted by equipment accuracy was no worse than ±1% and in temperature measuring bar ±0.5°C.

## Results and discussion

**Figure 2:**
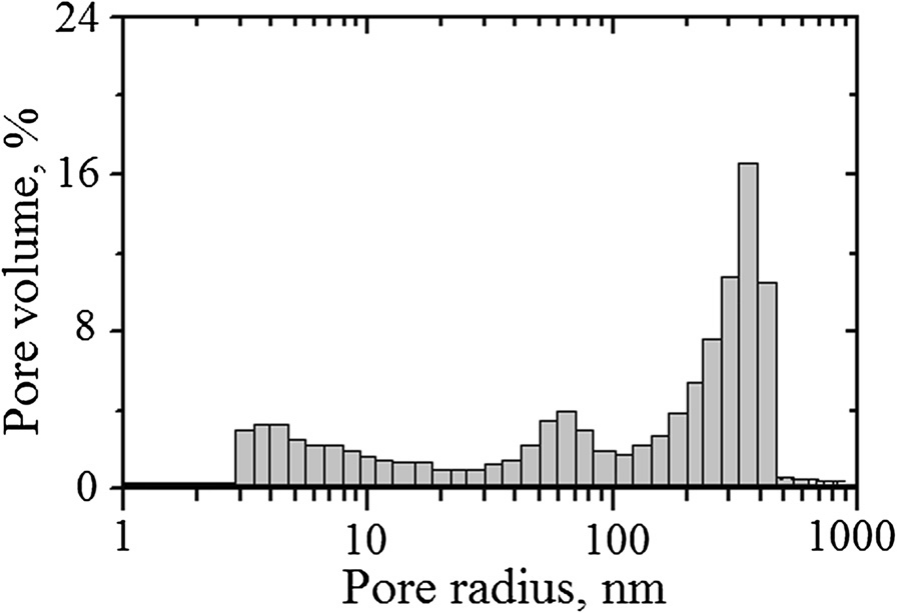
**Pore size distributions for humidity-sensitive MgAl**_**2**_**O**_**4 **_**ceramics sintered at 1,300°C for 5 h.**

Bulk dielectric MgAl_2_O_4_ ceramics, which are used for the preparation of humidity-sensitive thick-film layers, are characterized by tri-modal pore size distributions (Figure 2). This distribution covers the charge-transferring micro/nanopores (the first peak centered near 4 nm) depending on sintering conditions, water-exchange inside-delivering or communication mesopores (the second peak centered near 65 nm), and water-exchange outside-delivering macropores (the third peak centered near 350 nm) depending on the specific surface area of milled MgO-Al_2_O_3_ powder [24]. According to Kelvin equation [25], for capillary condensation processes of humidity in ceramics and their thick film at room temperature in the investigated range of RH (20% to 99%), the cylindrical pores with a radius from 1 to 20 nm are required. Meso- and macropores with radius more than 20 nm (the second and third peaks) are not involved in the capillary condensation process, but they ensure the effective transfer of water into ceramic bulk. Thus, the presence of pores in each area provides effective adsorption and desorption humidity processes in material bulk.

**Figure 3:**
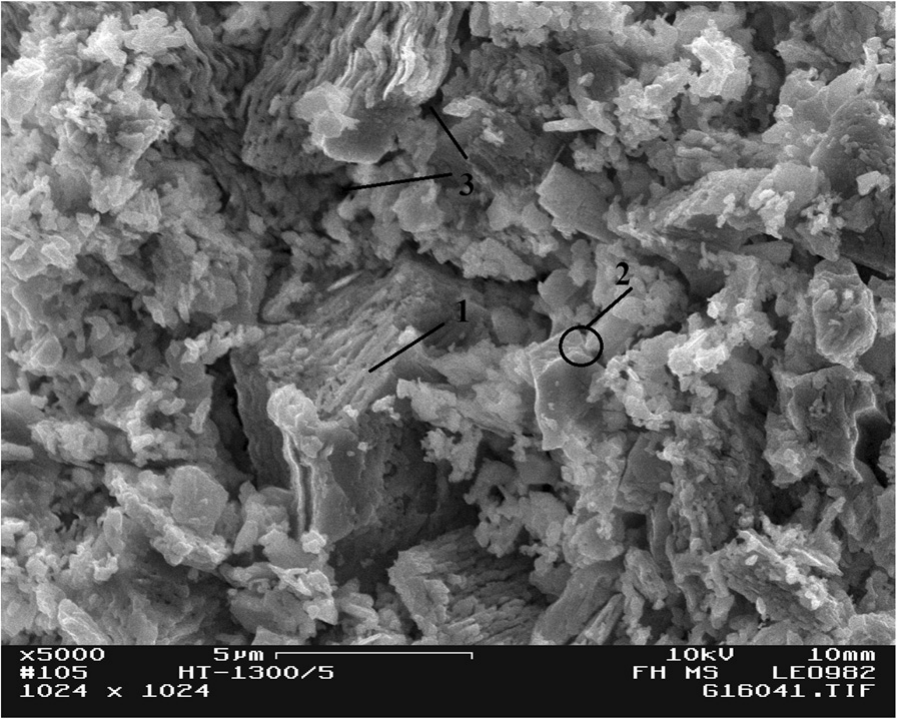
**SEM micrograph of MgAl**_**2**_**O**_**4 **_**ceramics sintered at 1,300°C for 5 h (1 - grain, 2 - grain boundaries, 3 - pore).**

As it follows from visual inspection of SEM images shown in Figure 3, the microstructure of humidity-sensitive ceramics is characterized by grains, grain boundaries, and pores. The grains are integrated into agglomerates. Spherical and cylinder pores are located near the grain boundaries. Average grain size for these ceramics is approximately 300 - 500 nm.

**Figure 4:**
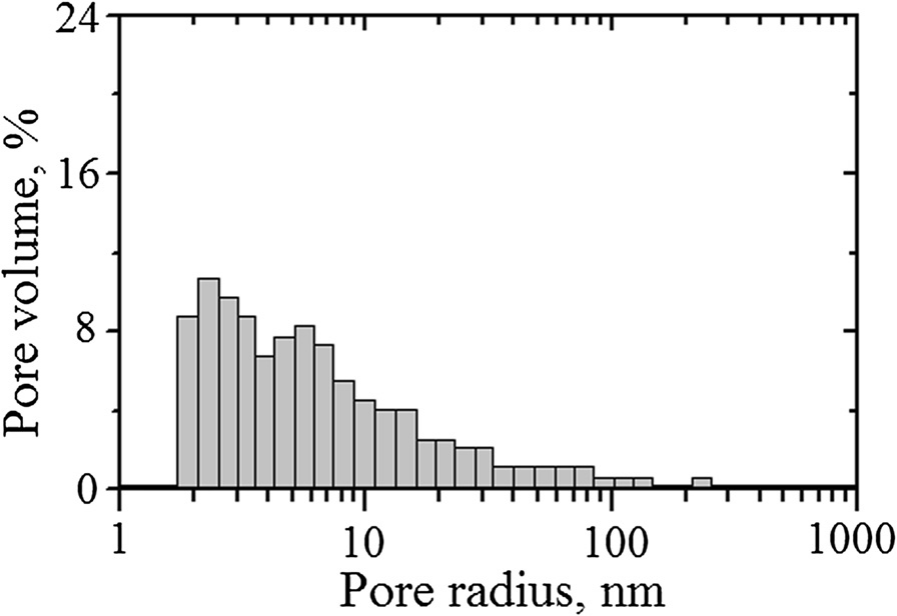
Typical pore size distributions for temperature-sensitive ceramics.

Typical pore size distribution for temperature-sensitive bulk ceramics are shown in Figure 4. It differs significantly from the pore size distribution for humidity-sensitive ceramics. This distribution covers only charge-transferring pores centered near 3.5 and 5.5 nm. But the amount of such pores is higher in comparison with MgAl_2_O_4_ ceramics.

**Figure 5:**
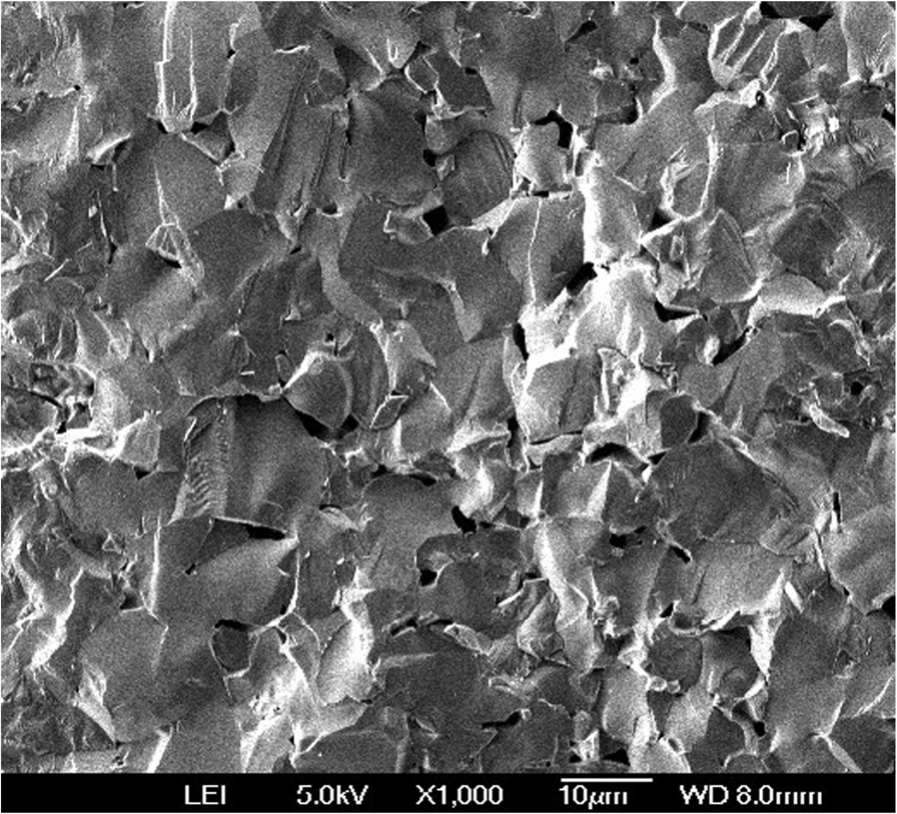
**Morphological structure of Cu**_**0.1**_**Ni**_**0.8**_**Co**_**0.2**_**Mn**_**1.9**_**O**_**4 **_**ceramics.**

In respect to the SEM data, the microstructure of temperature-sensitive ceramics is characterized by separate pores with 1 to 3 μm in sizes (Figure 5). White NiO film appears as bright layer of 10-μm thickness on the grain surface of these samples. The grain structure of ceramics attains monolithic shape. Individual pores of relatively large sizes (near 3 to 5 μm) are observed in these ceramics, the NiO appearing as uniform layer on the whole ceramic surface. The observed additional NiO phase is non-uniformly distributed within ceramic bulk, being more clearly pronounced near the grain boundaries [12].

These examined samples of temperature and humidity-sensitive ceramics with best microstructural and electrical properties have been used as base materials for the preparation of thick-film structures.

**Figure 6:**
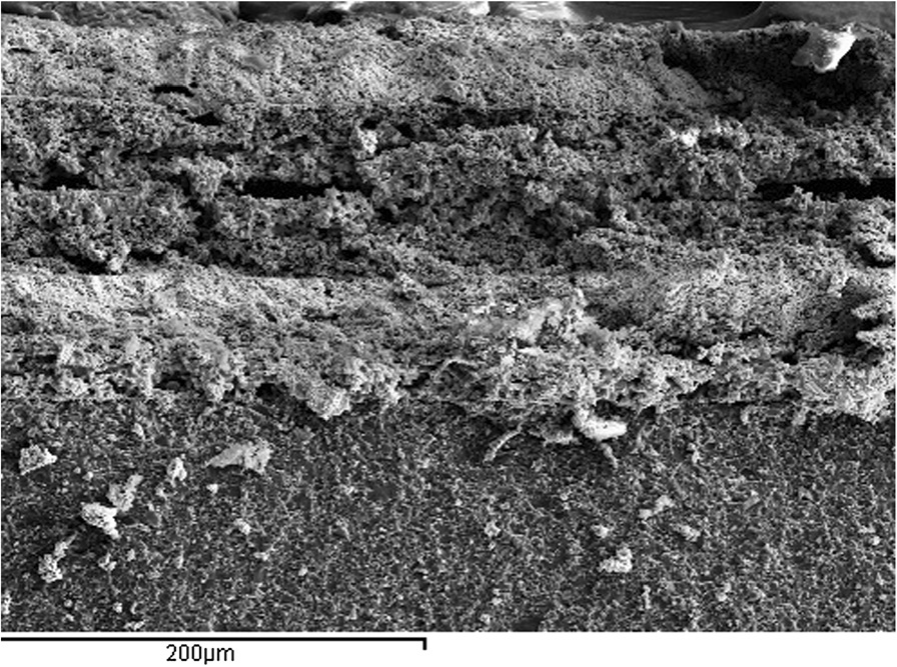
SEM micrograph of thick films prepared on alumina substrate.

The SEM micrograph of integrated p-i-p^+^ thick-film structure based on p^*+*^-type Cu_0.1_Ni_0.1_Mn_1.2_Co_1.6_O_4_ and p-type Cu_0.1_Ni_0.8_Mn_1.9_Co_0.2_O_4_ ceramics is presented in Figure 6. Micrograph reveals grains of basic ceramics, surrounded (‘covered’) by glass and pores. Thick films show higher density and microstructure homogeneity with uniform distribution of grains, glass additives, and pores. Contacting area of partially removed and peeled thick-film layers is evident from this micrograph. During the sintering process of thick-film structures, the diffusion of elements occurs from one layer into the near-surface region of the next layer with other conductivity [23]. Novel in this work is using p^*+*^-conductive Cu_0.1_Ni_0.1_Mn_1.2_Co_1.6_O_4_ layers to the preparation of contact area for humidity-sensitive i-type layers (see Figure 1). Such approach eliminates diffusion processes in the contact element material to thick films. So, we not only prepared an integrated multilayer p-i-p^+^ structure but also increased the active adsorption-desorption surface area for humidity-sensitive thick-film layers using the same spinel material not only as a temperature-sensitive layer but also as a conductive layer.

In spite of the same chemical type (spinel-like) of each thick-film layers, such effects correspond to the changes in their sensitivity, in particular, decreasing of sensitivity on i-type thick-film layer, due to diminishing of pores connected with capillary condensation processes [15] and additional phases near the grain boundaries [14].

**Figure 7:**
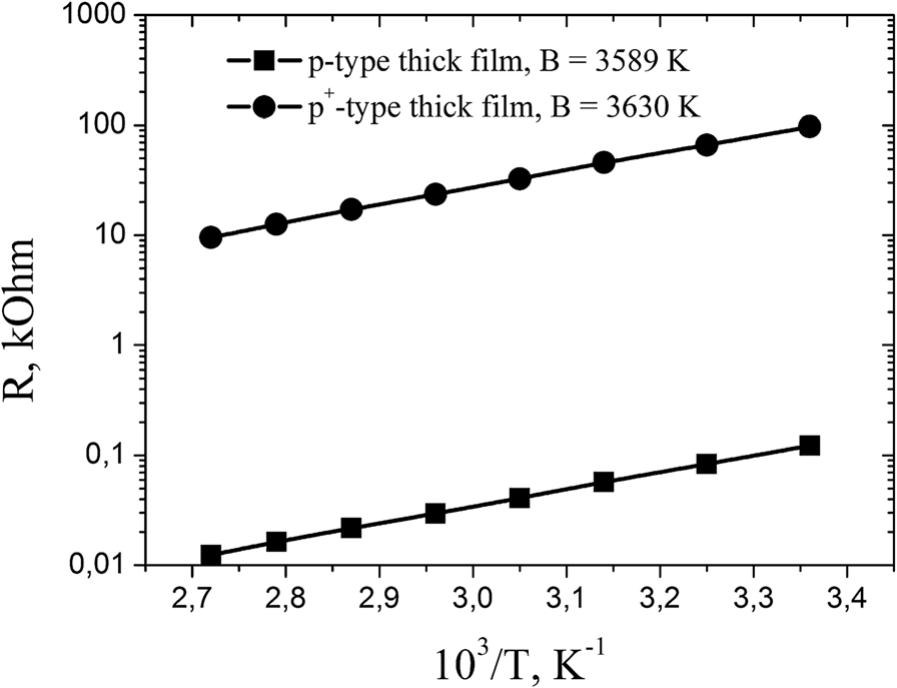
**Dependences of electrical resistance *R *** **on temperature for double p- and p**^**+**^**-conductive thick-film layers.**

All obtained p- and p^*+*^-conductive temperature-sensitive thick-film elements based on spinel-type NiMn_2_O_4_-CuMn_2_O_4_-MnCo_2_O_4_ ceramics have good electrophysical characteristics. These thick-film elements show linear temperature dependences of resistances (Figure 7). The values of *B*_25/85_ constant were 3,589 and 3,630 K for p-type Cu_0.1_Ni_0.8_Mn_1.9_Co_0.2_O_4_ and p^*+*^-type Cu_0.1_Ni_0.1_Mn_1.2_Co_1.6_O_4_ thick films, respectively. Both thick films possess good temperature sensitivity in the region from 298 to 358 K.

**Figure 8:**
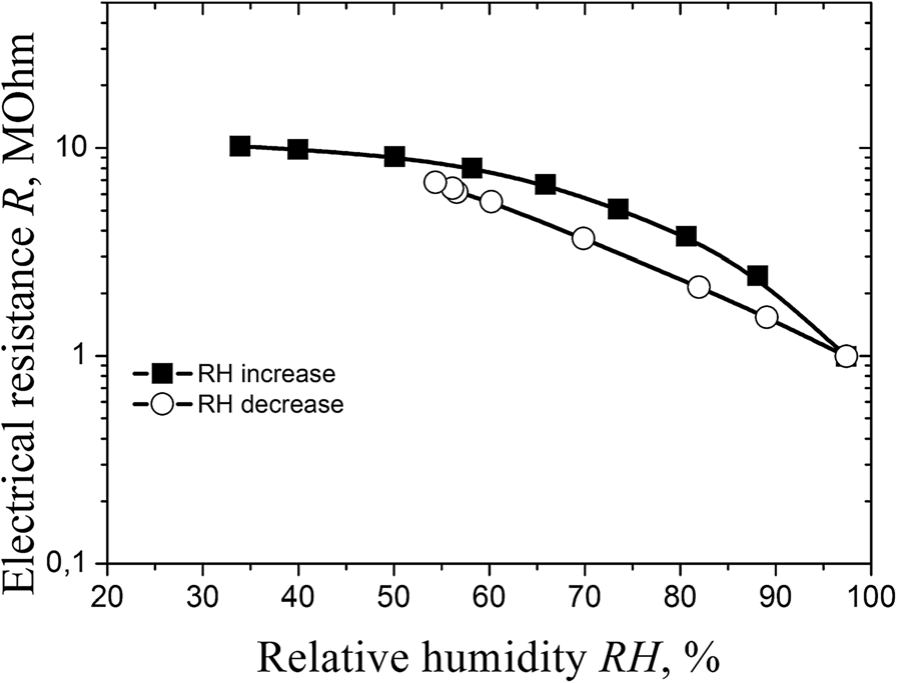
**Initial exploitation properties of integrated thick film p-i-p**^**+ **^**structures.**

**Figure 9:**
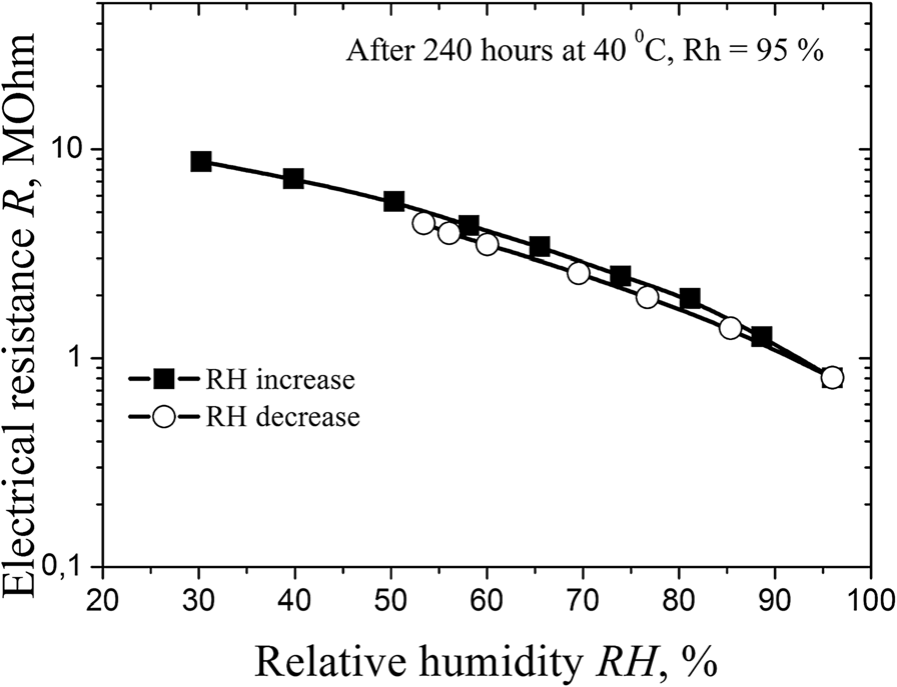
**Exploitation properties of integrated p-i-p**^**+ **^**thick-film structures after degradation transformation at 40°C and RH = 95% for 240 h.**

The studied thick-film elements based on i-type MgAl_2_O_4_ ceramics possess linear dependence of electrical resistance on RH in semilogarithmic scale with some hysteresis in the range of RH ~ 60% to 99% (see Figure 8). But after degradation transformation at 40°C for 240 h, the hysteresis is minimized (Figure 9). This effect corresponds to saturation of some nanopores of water, which provide effective adsorption-desorption processes [24]. Thus, these thick-film elements are suitable for humidity sensors working in the most important range of RH. The change of electrical resistance in p-i-p^+^ thick-film structures is 1 order, but these elements are stable in time.

Since all components are of the same chemical type (spinel-like) and possess high temperature/humidity sensitivities, they will be positively distinguished not only by wider functionality (simultaneous temperature-humidity sensing) but also by unique functional reliability and stability. In the case under consideration, the main advantages proper to bulk transition-metal manganite ceramics (wide range of electrical resistance with high temperature sensitivity) and humidity-sensitive MgAl_2_O_4_ ceramics will be transformed into thick-film multilayers, resulting in a principally new and more stretched functionality.

## Conclusion

Integrated temperature-humidity sensitive thick-film p-i-p^+^ structures with optimal grain-pore structures, where p^+^-conductive layers was used as a conductive layer, were obtained and studied. Temperature-sensitive thick-film structures possess good temperature sensitivity in the region from 298 to 358 K. The humidity-sensitive elements possess linear dependence of electrical resistance on relative humidity in semilogarithmic scale with some hysteresis in the range of RH ~ 60% to 99%. After degradation transformation, the hysteresis is minimized due to saturation of some nanopores by water, which provide effective adsorption-desorption processes in elements.

## Competing interests

The authors declare that they have no competing interests.

## Authors’ contributions

HK performed the experiments to study the temperature and humidity effects in thick-film structures and drafted, wrote, and arranged the article. IH proposed an idea of the development of integrated thick-film structures and performed the experiments to obtain bulk spinel ceramics and thick films. OS is a supervisor of the whole work, the results of which are presented in this article. MB supervised the experiments performed by IH. All authors read and approved the final manuscript.
